# Genome-Wide Analysis of *HIPP* Gene Family in Maize Reveals Its Role in the Cadmium Stress Response

**DOI:** 10.3390/genes16070770

**Published:** 2025-06-30

**Authors:** Chunyan Gao, Zhirui Zhang, Yuxuan Zhu, Jiaxin Tian, Kaili Yu, Jinbo Hou, Dan Luo, Jian Cai, Youcheng Zhu

**Affiliations:** 1 Biology and Food Engineering College, Fuyang Normal University, Fuyang 236037, China; gcy110@fynu.edu.cn (C.G.); fydiano@outlook.com (Z.Z.); 18221283435@163.com (Y.Z.); wl3467583975@163.com (J.T.); 13731815383@126.com (J.H.); 202406021@fynu.edu.cn (D.L.); 2 Funan Rural Revitalization Collaborative Technology Service Center, Fuyang Normal University, Fuyang 236037, China; 3 State Key Laboratory of Crop Genetics and Germplasm Enhancement and Utilization, Nanjing Agricultural University, Nanjing 210095, China; 2020216004@stu.njau.edu.cn

**Keywords:** maize, HIPP gene family, Cd, phytoremediation, expression pattern

## Abstract

Background: Phytoremediation is an efficient approach for remediating heavy metal-contaminated soils. Heavy metal-associated isoprenylated plant proteins (HIPPs)—crucial for metal ion homeostasis—are unique to vascular plants, featuring a heavy metal-associated (HMA) domain and an isoprenylated CaaX motif. However, *ZmHIPP* genes have not been systematically or functionally characterized in maize. Methods: This study characterizes *ZmHIPP* at the genome-wide level, including phylogenetic classification, motif/gene structure, chromosome location, gene duplication events, promoter elements, and tissue expression patterns. Cadmium (Cd) responses were evaluated by specific *ZmHIPP* expression and Cd accumulation in shoots and roots under Cd treatment. Results: A total of 66 *ZmHIPPs* were distributed unevenly across ten chromosomes, classified into five phylogenetic groups phylogenetically. Gene collinearity revealed 26 pairs of segmental duplications in *ZmHIPPs*. Numerous synteny genes were detected in rice and sorghum, but none in *Arabidopsis*, suggesting high conservation of *HIPP* genes in crop evolution. Transcriptomic analysis revealed tissue-specific expression patterns of *ZmHIPP* members in maize. Cis-acting element analysis linked several binding elements to abscisic acid, MeJA response, and MYB and MYC transcription factors. Under Cd stress, 53 out of 66 *ZmHIPP* genes were significantly induced, exhibiting three expression patterns. Cd exposure confirmed that the expression of *ZmHIPP11*, *ZmHIPP30*, and *ZmHIPP48* was generally higher in shoots than roots, while *ZmHIPP02* and *ZmHIPP57* exhibited the opposite. Cd accumulation was higher in roots than shoots, peaking at 72 h (96 mg/kg) in shoots and exceeding 1000 mg/kg in roots after 120 h. Conclusions: This study not only provides fundamental genetic and molecular insights into HIPP function in maize but also identifies specific *ZmHIPP* genes as promising genetic resources for breeding Cd-tolerant maize, aiding in phytoremediation of Cd-contaminated soils.

## 1. Introduction

Recently, soil contamination by heavy metals has increased mainly due to human activities, such as the direct discharge of industrial waste residue, fossil fuel combustion, extensive use of fertilizers and pesticides, and improper handling of urban domestic waste. This pollution inhibits plant growth, reducing crop yield and quality. Hazardous heavy metals, including cadmium (Cd), arsenic (As), copper (Cu), and lead (Pb), accumulate in crops, threatening human and animal health. Cd, a non-essential and highly toxic inorganic pollutant with high soil mobility and relative non-degradability, is particularly concerning [1]. Even trace amounts of Cd are toxic to plants, reducing enzyme activity, denaturing proteins, altering RNA synthesis, suppressing DNA repair, and inducing oxidative stress, culminating in cell or plant death [2].

Plants counter Cd stress through various mechanisms, including binding of Cd to cell walls and root exudates, suppression of root-to-shoot translocation via phloem restriction, Cd sequestration into vacuoles, and chelator-mediated Cd complexation for detoxification [3]. These processes are orchestrated via interconnected regulatory networks. Heavy metal-associated proteins (HMPs) in plants contribute to metal ion absorption, transport, and distribution, mitigating stress caused by their accumulation in cells [4]. HMPs include heavy metal-associated plant proteins (HPPs) and heavy metal-associated isoprenylated plant proteins (HIPPs), distinguished by a conserved CaaX motif (C = cysteine; a = aliphatic amino acid; X = any amino acid) at the C-terminus of HIPPs [5]. The CaaX motif is essential for carboxyl-terminal isoprenylation, a typical post-translational modification of many regulatory proteins. It binds proteins via hydrophobic side chains, facilitating specific interactions with the endomembrane system or other transport proteins. HIPPs contain at least one heavy metal-associated (HMA) domain (pfam00403) that sequesters heavy metals to prevent cytotoxicity [6].

HIPPs were first identified in *Arabidopsis* and are exclusive to vascular plants. They are crucial for plant development and environmental adaptation, and extensively participate in regulating responses to pathogens and abiotic stresses, including heavy metal toxicity [7]. However, research on HIPPs has focused primarily on *Arabidopsis* and rice, with limited functions explored. In *Arabidopsis*, the transcription factor AtMYB49 interacts with the promoters of *AtHIPP22* and *AtHIPP44*, enhancing their transcription and mediating subsequent Cd accumulation [8]. Heterologous expression of *AtHIPP20* and *AtHIPP22* restores Cd tolerance in *Δycf1* yeast mutants, whereas *AtHIPP21* does not confer similar resistance. The *hipp20/21/22* triple mutant in *Arabidopsis* exhibits increased Cd sensitivity and accumulates less Cd than the wild type [9]. *AtHIPP06* is induced by Cd and Cu, and its HMA domain in AtHIPP06 can bind Cd and Cu. *AtHIPP06* overexpression enhances Cd tolerance in yeast and *Arabidopsis* [10]. In rice, *OsHIPP29* [11] and *OsHIPP56* [12] are induced by Cd stress in roots. Overexpressing individuals exhibit enhanced Cd tolerance and reduced Cd content, while mutants display the opposite trend. This suggests that *OsHIPP29* and *OsHIPP56* mitigate Cd toxicity by decreasing its accumulation. Meanwhile, overexpression of *OsHIPP24* enhances Cu and Cd accumulation in the wild-type yeast BY4741; however, overexpression and knockout *OsHIPP24* lines exhibit stunted growth with reduced plant height in rice [13]. Cd, Mn, and Cu induce the expression of *OsHIPP42* and *OsHIPP34* in roots, as well as *OsHIPP16* and *OsHIPP28* in whole plants. Individual overexpression of *OsHIPP42*, *OsHIPP34*, or *OsHIPP60* specifically enhances yeast resistance to Cd, Cu, and Zn, respectively [4,14,15]. In maize, *ZmHIPP21*, *ZmHIPP09*, and *ZmHIPP02* are induced by Cd stress in roots [16]. Beyond heavy metal stress, HIPPs also aid in plant adaptation to other abiotic stresses, including cold and drought, as demonstrated by the inducible expression of *AtHIPP26* in *Arabidopsis* and *OsHIPP41* in rice. In terms of biotic stress, *AtHIPP27* is a host susceptibility factor for sugar beet cyst nematode infection, with reduced susceptibility in *AtHIPP27* knockout mutants [17].

To date, 59, 45, 45, 23, 56, and 26 *HIPP* genes have been identified in *Oryza sativa*, *Arabidopsis thaliana*, *Sorghum bicolor*, *Medicago sativa*, *Camellia sinensis*, and *Citrus sinensis*, respectively. These *HIPPs* are categorized into five clusters [5,9,18,19,20,21]. However, systematic genome-wide identification and functional characterization of the *HIPP* gene family remain unexplored in maize (*Zea mays* L.), a globally vital staple crop with significant importance in Cd accumulation and phytoremediation potential. Given the critical role of HIPPs in heavy metal detoxification and their differential expression patterns under Cd stress, it is plausible that specific *ZmHIPP* members in maize may function as key regulators of Cd tolerance and accumulation. In the current study, the *ZmHIPP* gene family was characterized in maize at the genome-wide level. The analysis of phylogeny, motif/gene structure, and gene duplication events indicates that the *ZmHIPP* gene family has undergone evolutionary expansion to enhance functional diversity under Cd stress. Cis-acting elements analysis suggests stress-responsive elements in *ZmHIPP* promoters help drive spatiotemporal expression during Cd exposure. Additionally, expression patterns in leaves and roots at multiple time points under Cd stress and the corresponding Cd content were examined to define the relationship between *ZmHIPP* expression and Cd accumulation. Collectively, the results of this study provide a basis for elucidating the biological functions of *ZmHIPP* genes in response to Cd stress and identify potential targets for developing Cd-tolerant maize.

## 2. Materials and Methods

### 2.1. Plant Materials and Experimental Treatments

The maize (*Z. mays* inbred B73) plants were cultivated in a culture chamber at 30 °C/25 °C under a 16 h light/8 h dark photoperiod. Maize seedlings were subjected to Cd stress via application of 200 mg/L CdCl_2_ at ten days post-germination. The shoots and roots were sampled separately at 0, 12, 24, 48, 72, and 120 h after the onset of Cd exposure. Samples were rapidly frozen and stored at −80 °C pending further analyses.

### 2.2. RNA Isolation and Real-Time RT-PCR Analysis

Total RNA from maize shoots and roots was isolated using the RNAiso Plus reagent (Takara). The upper extract was precipitated by isopropanol and washed twice with 75% ethanol. The first-strand cDNA was synthesised in one step with a reagent kit (*TransScript*^®^ All-in-One First-Strand cDNA Synthesis SuperMix) from TransGen Biotech Co., Ltd., Beijing, China.

Expression analysis of *ZmHIPPs* transcripts in response to Cd stress was conducted by quantitative real-time PCR (qRT-PCR) in both shoots and roots. cDNA templates were amplified employing the ChamQ Universal SYBR qPCR Master Mix (Vazyme, Nanjing, China). PCR amplifications were carried out on a BIO-RAD CFX Connect instrument under the following cycling conditions: 40 cycles of denaturation at 95 °C for 10 s, annealing at 58 °C for 10 s, and extension at 72 °C for 30 s. Each cDNA sample was assayed in triplicate. Relative gene expression levels were calculated using the 2^−ΔΔCt^ method. *ZmTUB* served as the reference gene for normalization. The sequences of all primers used are provided in Table S1.

### 2.3. Identification and Physicochemical Properties of HIPP Genes in Maize

Genome sequences, protein sequences, and genome annotation of maize were obtained from the Ensembl database. The 45 Arabidopsis and 59 rice HIPP protein sequences were obtained from the Arabidopsis Information Resource database and Rice Genome Annotation Project, respectively. The OsHIPP protein sequences were used as a reference to obtain the homologous genes from maize using TBtools software (v2.310). The hidden Markov model (HMM) of HMA (PF00403) was also obtained from the Pfam database, and then the maize protein sequences were searched using TBtools. The data obtained via BLAST (version 2.14.0+) and HMM analyses were compared, and duplicate genes were removed. The candidate ZmHIPPs were screened according to whether the proteins contained the isoprenylation motif CaaX at the C-terminus. The amino acid number, molecular mass, theoretical pI, instability index, aliphatic index, and grand average of hydropathicity (GRAVY) of ZmHIPP proteins were computed via the ExPASy online platform (https://www.expasy.org/, accessed on 10 February 2025). Additionally, predictions regarding the subcellular localization of ZmHIPPs were generated using the WoLF PSORT web server.

### 2.4. Phylogenetic Analysis and Classification

The HIPP proteins of maize, Arabidopsis, and rice were compared using ClustalW with MEGA 7 software (version 7.0.26). The phylogenetic tree was constructed using the neighbor-joining method in MEGA7. The bootstrap value was set at 1000. The draft was optimized using the Interactive Tree of Life website (https://itol.embl.de/, accessed on 12 February 2025).

### 2.5. Conserved Motif, Domain, and Gene Structure Analysis of ZmHIPPs

The identified ZmHIPP proteins were analyzed for conserved motifs and functional domains using TBtools with motifs set at 10 and leaving the other parameters set to default. The conserved structural domains of ZmHIPPs proteins were analyzed using the Batch CD-search module from the National Center for Biotechnology Information. The Gene Structure View module from TBtools was used for visualization.

### 2.6. Chromosome Distribution and Synteny Analysis in ZmHIPP Gene Family

The chromosomal localization of the *ZmHIPP* gene family members within the maize genome was analyzed and visualized using GTF/GFF from TBtools. The genome sequences and the annotated files for maize, Arabidopsis, rice, and sorghum were retrieved from Ensembl Plants (https://plants.ensembl.org, accessed on 13 February 2025). Synteny analyses for maize, Arabidopsis, rice, and sorghum were performed using One Step MCScanX in TBtools. Gene duplication and collinearity were visualized using TBtools.

### 2.7. Analysis of ZmHIPP Cis-Acting Elements

The promoter sequences 2000 bp upstream of *ZmHIPP* genes were analyzed using TBtools, and the cis-acting elements were predicted using the online tool PlantCARE. Some elements, such as TATA or CAAT boxes, were excluded. A large number of repeated elements with related annotations were selected and visualized using the Simple BioSequence Viewer from TBtools.

### 2.8. Expression Profiling of ZmHIPPs

Based on the published transcriptomic data [22], the expression patterns of *ZmHIPPs* were analyzed across 23 tissues and organs at different developmental stages. The female spikelets and silk were collected on the same day. FPKM values were transformed by log_2_ (FPKM + 1) and then used to construct the heatmap. Previous researchers have conducted a transcriptomic analysis for ten-day maize seedlings exposed to 200 mg/L CdCl_2_ [23]. The aboveground parts of B73 seedlings were sampled at different time intervals (0 h, 12 h, and 72 h) after Cd treatment for RNA-seq analysis. The mean FPKM value of three biological replicates was calculated for any given gene. Genes were considered expressed if the average FPKM value was not less than 0.5. Thus, 25,907 genes that were expressed in at least one sample were obtained, and we screened the upregulated or downregulated *ZmHIPP* genes among these genes. Heatmaps were generated from the collected FPKM values of the control and treatment groups using the Lianchuan Bio online platform (https://www.omicstudio.cn/tool/, accessed on 15 February 2025).

### 2.9. Determination of Cd Concentration

To determine the Cd content in the aboveground parts and roots, dried samples were subjected to mixed acid digestion. First, 9 mL of HNO and 3 mL of HF were added to a digestion tube containing 0.20 g of dry sample. The tightly capped digestion tubes were placed into a hot plate, heated to 200 °C, and maintained for 4 h. After digestion, the tube lids were opened and placed at 150 °C for 2 h until approximately 1 mL remained. The mixture was then transferred to a 50-mL volumetric flask, supplemented with 0.50 mol/L nitric acid and 100 μg/L Au solution, and brought to volume (50 mL) with deionized water. Finally, determination of Cd content was performed using an inductively coupled plasma mass spectrometer (ICP-MS, Agilent7800; Agilent Technologies, Santa Clara, CA, USA).

## 3. Results

### 3.1. Characterization and Physicochemical Properties of HIPP Genes in Maize

**Figure 1:**
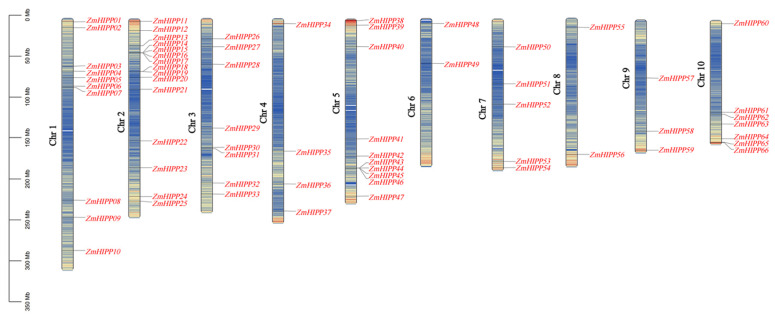
Distribution of the HIPP gene family on maize chromosomes. Genomic distributions of 66 *HIPP* genes across the 10 maize chromosomes. The physical coordinates are accurately depicted on the chromosome.

A total of 66 *ZmHIPPs* were identified based on the CysXXCys motif in the HMA domain and a C-terminal isoprenylation CaaX motif. These genes were designated *ZmHIPP1* to *ZmHIPP66* based on their chromosomal locations (Figure 1). Chromosomal localization analysis showed that the 66 *ZmHIPP* genes were unevenly distributed across the ten maize chromosomes, with significant enrichment in distal chromosomal regions. Chromosome 2 had the most *ZmHIPP* genes (15 genes), followed by Chromosomes 1 and 5 with ten *ZmHIPP* genes each. Chromosomes 3, 10, 7, 4, and 9 possessed eight, seven, five, four, and three *ZmHIPP* genes, respectively. Meanwhile, Chromosomes 6 and 8 each carried two *ZmHIPP* genes. This distribution pattern suggested no significant correlation between chromosome length and *ZmHIPP* gene density.

The lengths of proteins encoded by the 66 *ZmHIPPs* ranged from 99 to 551 amino acids, with corresponding molecular weights varying between 11.57 kDa (ZmHIPP35) and 55.21 kDa (ZmHIPP60; Table S2). The theoretical pI values varied from 5.00 (ZmHIPP13) to 9.83 (ZmHIPP8). Among the 66 ZmHIPP proteins, 49 were categorized as alkaline proteins (pI > 7). Instability coefficients ranged from 17.05 (ZmHIPP12) to 85.13 (ZmHIPP10); 19 proteins, including ZmHIPP1, ZmHIPP2, and ZmHIPP9, exhibited higher stability with coefficients below 40. The aliphatic index varied between 32.85 (ZmHIPP60) and 87.59 (ZmHIPP44). All proteins were hydrophilic, as indicated by negative GRAVY values. Predicted subcellular localizations revealed that most ZmHIPP proteins were in the cytoplasm (23), nucleus (20), and chloroplasts (19).

### 3.2. Phylogenetic Analysis of ZmHIPP Proteins Based on Conserved Motifs and Gene Structure

**Figure 2:**
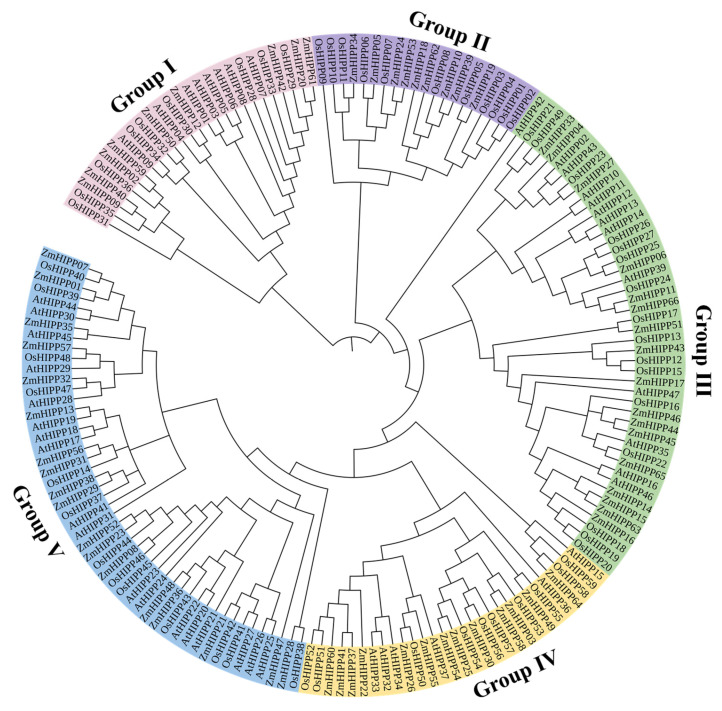
Phylogenetic analysis and classification of HIPPs in *Z. mays*, *A. thaliana,* and *O. sativa*.

Phylogenetic analyses can elucidate evolutionary relationships between homologous HIPP proteins across species. A phylogenetic tree was constructed using MEGA software (version 7.0) by combining 66 *ZmHIPPs* with 45 *AtHIPPs* and 59 *OsHIPPs* (Figure 2). Based on the topology of the phylogenetic tree, the *ZmHIPP* family was divided into five groups (I: 9, II: 9, III: 17, IV: 13, and V: 18 genes), consistent with rice and *Arabidopsis*. *ZmHIPP* members within each group exhibit close evolutionary relationships, implying similar functional roles.

**Figure 3:**
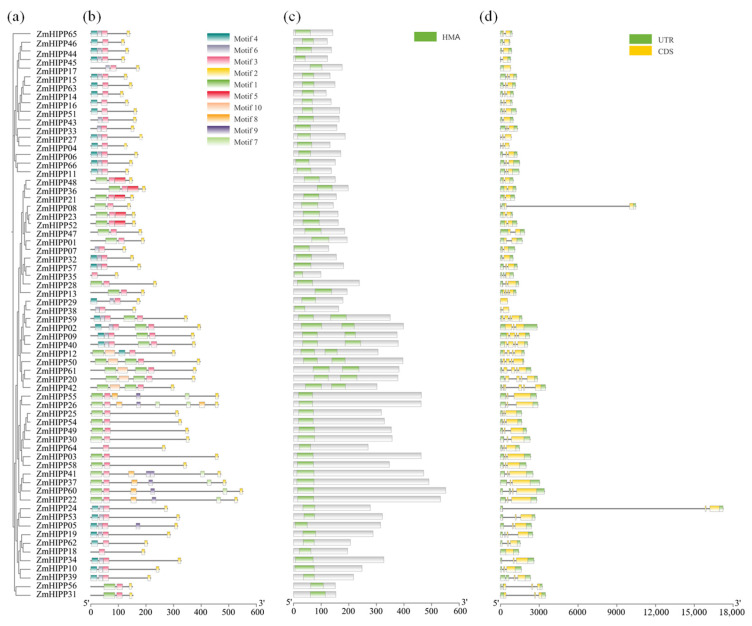
Analysis of the gene structure, conserved motifs, and protein-coding domains of the *HIPP* gene family in maize. (**a**) The phylogenetic tree was constructed using ClustalW based on the amino acid sequences of *HIPP* genes. (**b**) Composition and distribution of conserved motifs in the ZmHIPP proteins, with 10 motifs listed above. (**c**) Conserved domain of the *ZmHIPP* family genes. (**d**) Gene structure of the *ZmHIPP* gene family. Coding sequences and introns are depicted using yellow boxes and black lines, respectively. The *HIPP* gene family is divided into five groups based on characteristics of the above aspects.

MEME analysis of ZmHIPP identified ten conserved motifs; motifs 2 and 3 were in all ZmHIPPs. ZmHIPPs within a group shared similar motifs (Figure 3). Analysis of the amino acid sequences revealed that motif 3 was an HMA domain, while motif 2 was an isoprenylated CaaX motif at the C-terminus. Nine ZmHIPPs in group I contained two HMA domains, whereas the remaining ZmHIPPs contained one HMA domain. HMA domains enable HIPPs to fold βαββαβ secondary structures for binding heavy metals, maintaining metal homeostasis in cells. The isoprenylation site affects HIPPs’ localization and interactions [19]. Additionally, *ZmHIPPs* in the same group displayed similar exon/intron structures. *ZmHIPPs* in group I had four or six exons, more than the other groups (one to four exons). *ZmHIPP08* and *ZmHIPP24* contained a particularly long intron. Additionally, group IV *ZmHIPPs* were longer, while those in group III were the shortest.

### 3.3. Synteny Analysis in HIPP Genes

**Figure 4:**
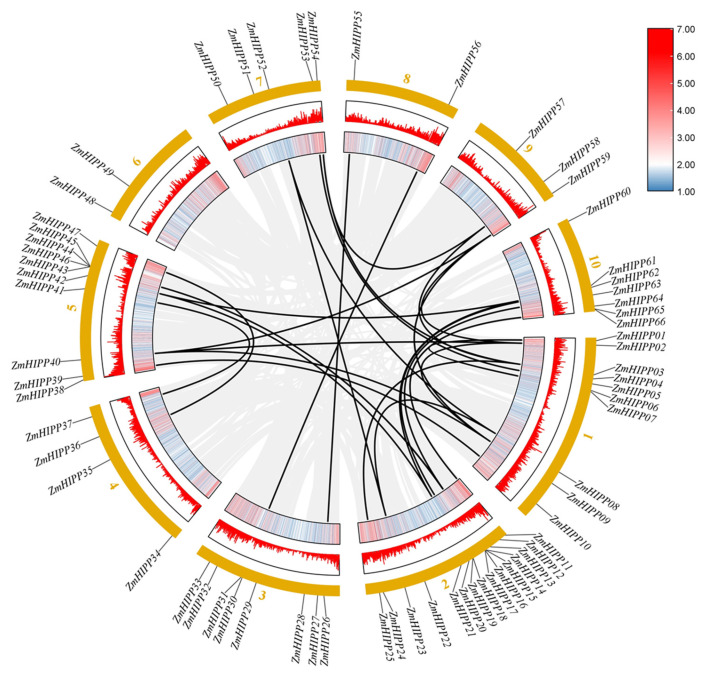
Intraspecific synteny analysis in the *HIPP* gene family of maize. The middle grey lines indicate all collinear blocks in the maize genome as background. The red lines represent duplicated *HIPP* gene pairs in maize.

**Figure 5:**
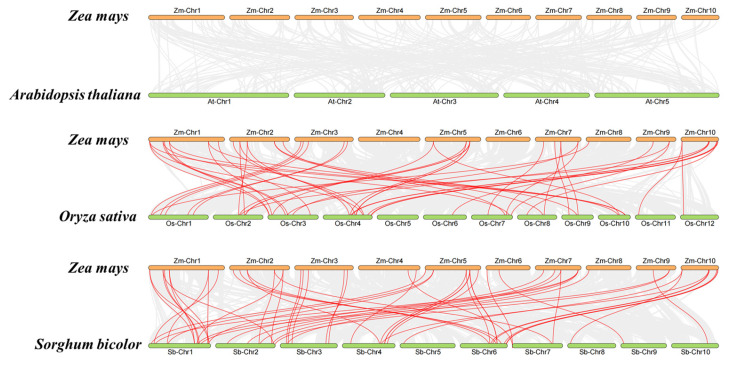
Synteny analysis in the *HIPP* gene family of *Z. mays*, *S. bicolor*, *A. thaliana,* and *O. sativa.* Gray lines in the background present all synteny modules in the genome, and the blue lines indicate the collinearity module of the *HIPP* gene pairs across different species.

Gene duplications generate new genes throughout evolution. Given the importance of tandem and segmental duplication events in gene families, *ZmHIPP* gene duplication events were investigated, and 26 segmental duplication events were identified. As shown in Figure 4, four groups of tandemly duplicated genes were detected in the *ZmHIPP* gene family: (1) *ZmHIPP14* and *ZmHIPP15*, (2) *ZmHIPP16* and *ZmHIPP17*, (3) *ZmHIPP18* and *ZmHIPP19*, and (4) *ZmHIPP43, ZmHIPP44,* and *ZmHIPP45*. Additionally, the maize *HIPP* members contained 56 and 51 collinear gene pairs with the monocots sorghum and rice, respectively. However, no synteny genes were detected in dicot *Arabidopsis* (Figure 5). This suggests that *ZmHIPP* genes may have originated from monocot divergence.

### 3.4. Analyses of ZmHIPP Gene Promoter Cis-Regulatory Elements

**Figure 6:**
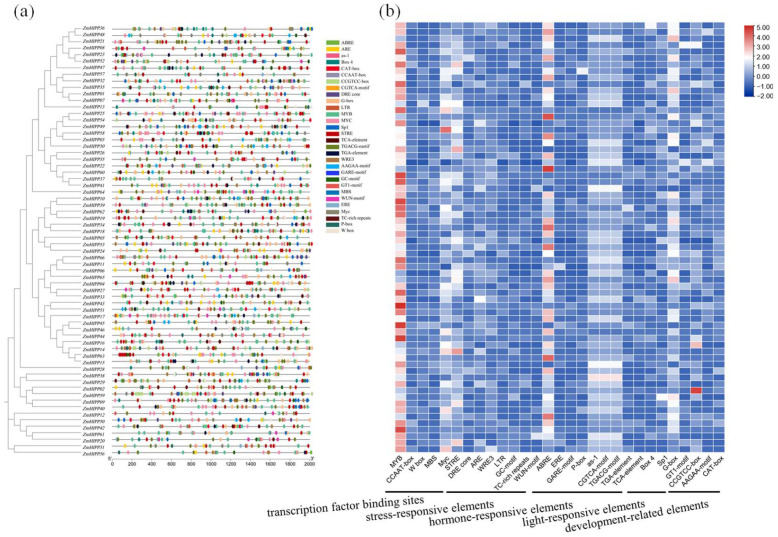
Cis-acting elements analysis in the promoter region of the *ZmHIPP* gene family. The figure presents the main types of cis-acting elements and their locations in the 2000 bp upstream region of the *ZmHIPP* transcription start site. (**a**) The distribution of cis-acting elements on the promoter. (**b**) Statistics on the number of cis-acting elements classified.

To investigate the transcriptional regulation of *ZmHIPPs* in maize, their promoters were analyzed by examining the 2000 bp region upstream of the transcription start site and predicting cis-acting regulatory elements using PlantCARE (Figure 6). A total of 3290 cis-elements were annotated: 905 transcription factor binding sites, 596 stress-responsive elements, 1097 hormone-related elements, 450 light-responsive elements, and 242 plant growth-associated elements. Notably, each *ZmHIPP* contained 5–9 MYB-binding elements. Massive biotic and abiotic stress-related elements were presented in *ZmHIPPs*, including the stress response promoter element (STRE), GC-motif (related to anoxic-specific inducibility), anaerobic induction element (ARE), dehydration-responsive element (DRE), and wound-responsive element (WRE3). Cis-acting elements that respond to MeJA, such as the TGACG-motif and CGTCA motifs, were prevalent, alongside other hormone-related elements like ABRE, as-1, TGA, and P-box—related to abscisic acid, salicylic acid, auxin, and gibberellin, respectively. Light-responsive elements included BOX 4, Sp1, G-box, and GT1-motif, whereas growth-associated elements comprised CCGTCC motif, AAGAA motif, CAT-box (related to meristem expression), and RY element (related to seed-specific regulation). Collectively, cis-acting element analysis of the *ZmHIPP* gene family revealed numerous elements related to light responsiveness, hormone and stress responsiveness, and growth and development, suggesting that *ZmHIPPs* play integrated roles in stress response and growth regulation.

### 3.5. Expression Profiling of ZmHIPP Genes in Maize

**Figure 7:**
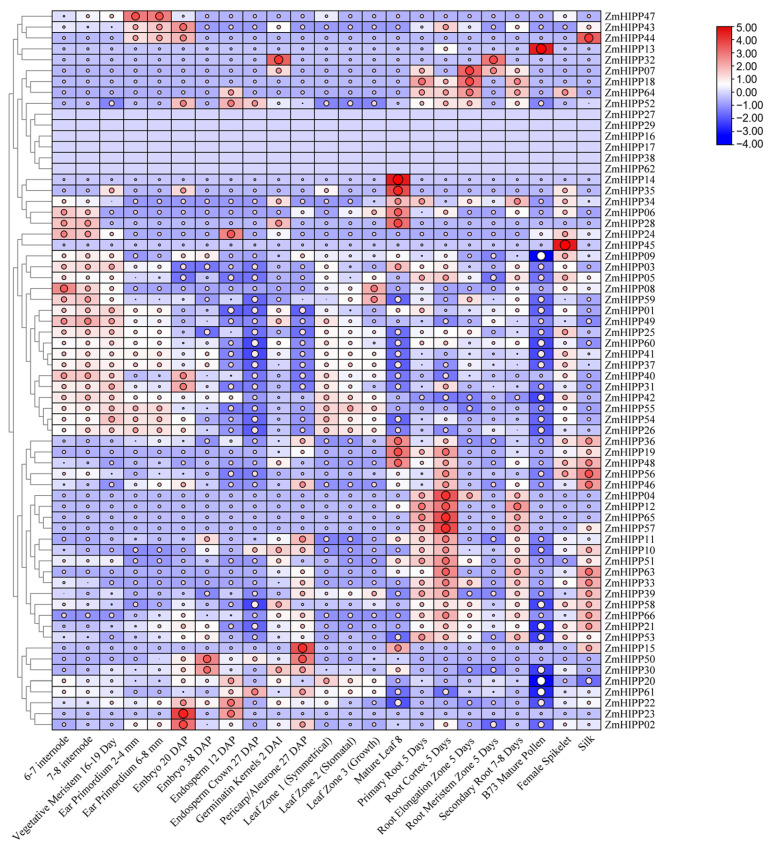
Analysis of the expression patterns of *ZmHIPP* genes across different tissues and organs. The abundance of transcript levels is represented by a color gradient from red to blue. “NA” (not available) was replaced by FPKM = 0. The values of log_2_ (FPKM + 1) are visualized as a heatmap.

To investigate the tissue-specific expression patterns of *ZmHIPPs*, transcript abundances were analyzed in 23 maize tissues and organs. The clustering heatmap revealed that the expression of *ZmHIPP04*, *ZmHIPP07*, *ZmHIPP11*, *ZmHIPP12*, *ZmHIPP18*, *ZmHIPP51*, *ZmHIPP57*, *ZmHIPP64*, and *ZmHIPP65* was significantly higher in roots than in other tissues (Figure 7). Meanwhile, *ZmHIPP06*, *ZmHIPP14*, *ZmHIPP19*, *ZmHIPP28*, *ZmHIPP34*, *ZmHIPP35*, *ZmHIPP36*, and *ZmHIPP48* were upregulated in leaves compared with the roots and stems. Additionally, *ZmHIPP02* and *ZmHIPP23* were predominantly expressed in embryos, while *ZmHIPP13*, *ZmHIPP44*, *ZmHIPP45,* and *ZmHIPP47* were highly expressed in other reproductive organs, including pollen, silk, spikelets, and ears, respectively. Overall, *ZmHIPP* family members exhibit distinct tissue-specific expression patterns throughout the life cycle of maize.

### 3.6. ZmHIPP Expression Under Cd Stress and Corresponding Cd Accumulation in Maize

**Figure 8:**
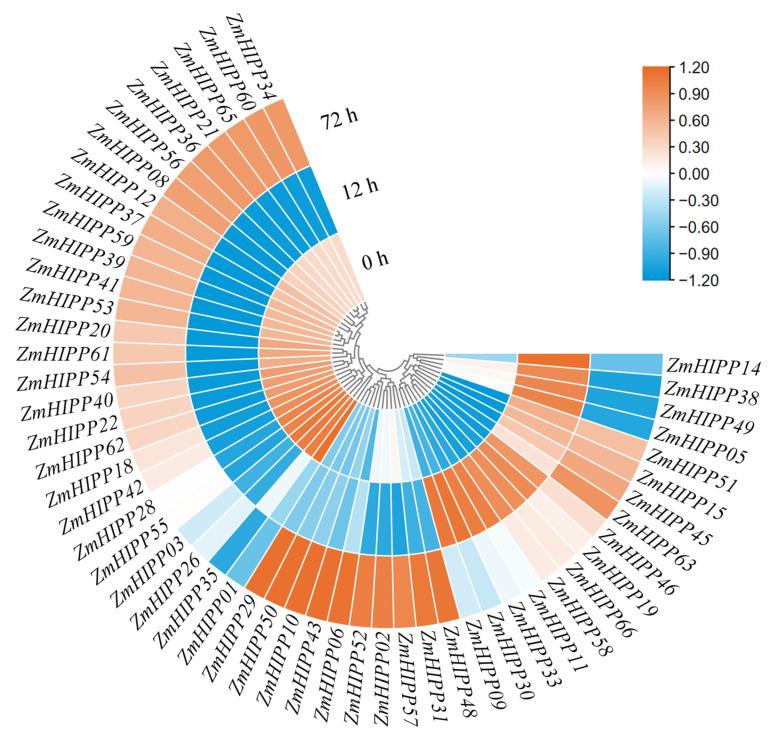
Expression profiles of *ZmHIPP* genes under Cd exposure (100 µM).

When maize seedlings were subjected to Cd stress, 53 of the 66 *ZmHIPP* genes were significantly induced at 12 h or 72 h (Figure 8). Three expression patterns emerged: (1) expression increased after 72 h of Cd stress (e.g., *HIPP02*, *HIPP48*); (2) responded within 12 h to Cd stress (e.g., *ZmHIPP11* and *ZmHIPP30*); (3) high expression under normal conditions, decreased after 12 h of Cd stress, and increased at 72 h (e.g., *ZmHIPP34* and *ZmHIPP40*).

**Figure 9:**
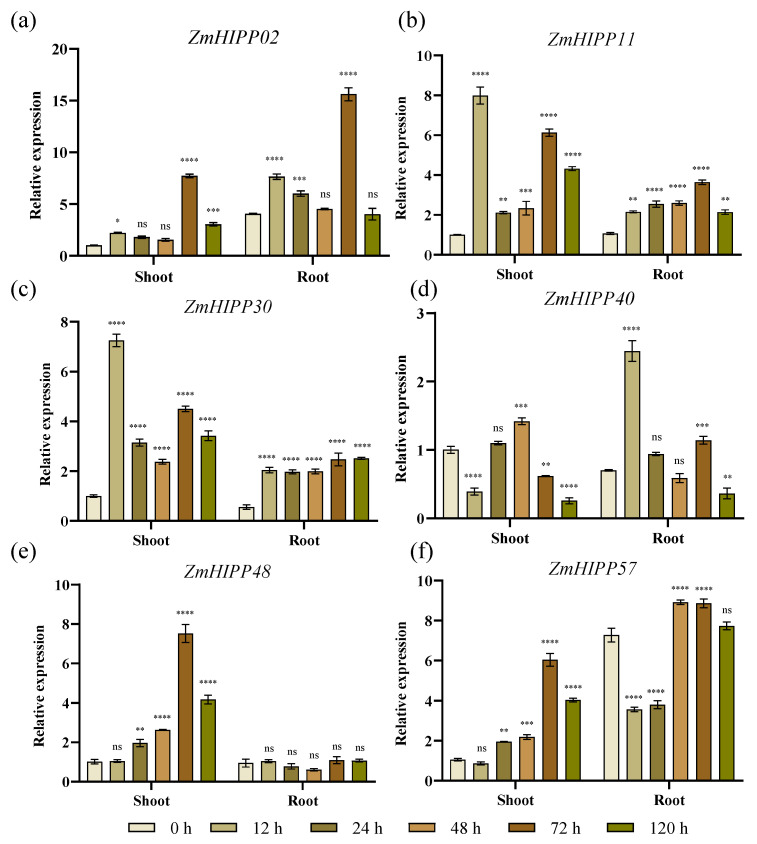
The expression changes of the *ZmHIPPs* gene family in maize under Cd stress were analyzed using qRT-PCR. (**a**) *ZmHIPP02*; (**b**) *ZmHIPP11*; (**c**) *ZmHIPP30*; (**d**) *ZmHIPP40*; (**e**) *ZmHIPP48*; (**f**) *ZmHIPP57*. Each experiment included three biological replicates and three technical replicates. Data are presented as the mean ± SD. Statistical significance is denoted as follows: “****” for *p* < 0.00001, “***” for *p* < 0.0001, “**” for *p* < 0.001, “*” for *p* < 0.05, and “ns” for no significant difference.

B73 plants were assessed under Cd stress to verify gene expression. Six *ZmHIPP* genes were selected for expression analysis in shoots and roots. The expression patterns of the six genes were consistent with the transcriptome data (Figure 9). The expression of *ZmHIPP11*, *ZmHIPP30*, and *ZmHIPP48* was generally higher in leaves than roots, while *ZmHIPP02* and *ZmHIPP57* showed the opposite pattern. *ZmHIPP48* was induced only in leaves. The expression of *ZmHIPP11* and *ZmHIPP30* in leaves rapidly increased within 12 h of Cd stress, similar to *ZmHIPP40* in roots.

**Figure 10:**
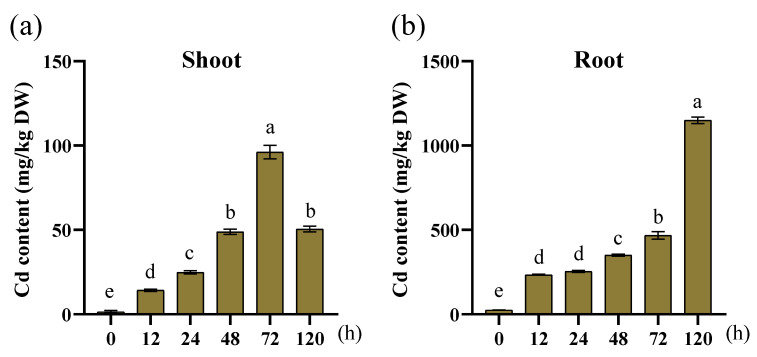
Cd accumulation in the shoot and root tissues of maize seedlings under Cd stress. (**a**) Cd content in the shoot tissue; (**b**) Cd content in the root tissue. Each experiment was conducted in triplicate, and the data are presented as the mean ± SEM. Statistical significance was determined using the Tukey multiple comparison test (*p* < 0.05).

Cd content was measured in the aboveground parts and roots of treated plants. Cd was transported from the soil to the shoots via root absorption. Cd accumulation was markedly higher in roots than leaves (Figure 10). At 12 and 120 h post-treatment, roots exhibited a significant increase in Cd accumulation. In shoots, Cd accumulation peaked at 72 h and decreased at 120 h. Cd toxicity caused yellowing of leaves in the later stage of treatment, as they exhibited a degree of tolerance. However, ultimately, Cd severely impaired plant growth.

## 4. Discussion

In the current study, genomic analysis identified 66 ZmHIPPs containing HMA domains and CaaX motifs, which is more than the majority of plants, including those recently identified in sorghum, alfalfa, and citrus [18,19,21]. Based on the conserved motifs and domains, ZmHIPP proteins were classified into five groups similar to the gene family in Arabidopsis and rice [24], with protein structures varying among groups. Group I of ZmHIPPs contained two HMA domains that facilitate binding to more metal ions, promoting metal ion uptake and maintaining metal ion homeostasis, similar to AtHIPP07 [25] and OsHIPP33 [26]. In contrast, the HIPPs in Group IV were relatively large and could bind heavy metal ions through HMA motifs, sequestering them in the cell wall. This prevents the penetration of heavy metals into other organelles, thus mitigating the toxicity of free heavy metal ions on cells, as reported for ZmHIPP22 [27].

Isoprenylation is a post-translational modification that covalently attaches hydrophobic groups to target proteins, allowing them to interact specifically with membrane and transporter proteins, thereby altering protein localization [28]. After entering the cells, metal ions are relocated via metal transporter proteins, such as movement from the cytoplasm to vacuoles [29]. ZmHIPP22 [27], SbHIPP40 [18], CsHIPP10, CsHIPP19, CsHIPP22 [21], OsHIPP16 [30], OsHIPP33 [26], OsHIPP29 [11], OsHIPP56 [12], and OsHIPP42 [14] are localized in the nucleus and plasma membrane, while CsHIPP19 and CsHIPP22 [21] are within the nucleus and microtubules, and TaHIPP1 [31] and AtHIPP3 [32] are specifically localized in the nucleus. Within the current study, most ZmHIPPs were predicted to be localized in the cytoplasm, nucleus, and chloroplasts. But this requires experimental confirmation.

In plants, gene duplication events such as tandem duplication and segmental duplication contribute significantly to genomic expansion, facilitating the emergence of new genes and evolutionary novelty [20]. Tandem duplication generates clustered gene families with high sequence homology and functional similarity on a single chromosome. Nine *ZmHIPP* genes (13.6%) are found to be tandem repeats in maize. There are four distinct pairs of tandemly duplicated genes, of which three pairs are located on Chromosome 2, and another group of 3 genes is located on Chromosome 5. Meanwhile, Chromosome 2 contains the most *ZmHIPP* genes (15), suggesting that this chromosome may be an evolutionary hotspot of the *ZmHIPP* family due to the local chromatin environment or selection pressure. A collinearity analysis of *ZmHIPP* family genes detects 26 segmental duplication events within 38 *ZmHIPPs*, suggesting that the abundance of chromosomal segment duplication may be a possible reason for the larger number of *ZmHIPPs*. Cross-species comparisons reveal that 40 and 34 *ZmHIPP* genes exhibit 56 and 51 collinear gene pairs with sorghum and rice, respectively. However, no collinear *HIPP* gene pairs are detected between maize and Arabidopsis. The exclusive presence of collinear *ZmHIPP* pairs in monocots, coupled with their absence in dicots, strongly supports their origin subsequent to the monocot-dicot divergence event during *HIPP* family evolution and suggests that HIPP genes might have evolved unique and essential functions in monocot environmental adaptation. Additionally, genes like *ZmHIPP53* exhibit multiple collinear relationships (2–3 gene pairs) between maize and sorghum/rice, suggesting their potential role as evolutionary hubs in the diversification of the *ZmHIPP* gene family.

The cis-acting element analysis of *ZmHIPP* genes identifies numerous stress-responsive elements and hormone-related elements, indicating that these genes might be involved in stress tolerance and growth regulation. Notably, we identify 905 (27.5%) cis-acting sites that potentially interact with transcription factors, such as MYB, MYC, and WRKY (W box). Transcription factors like WRKY [33,34], MYB [35], bHLH [36,37], and ERF [38] have been reported to participate in modulating gene expression to counteract Cd toxicity. Therefore, further prediction and analysis of the transcriptional regulation of *ZmHIPP* genes are necessary, and the binding sites of MYB or WRKY represent preferred targets for screening the core promoter motifs of *ZmHIPP* genes.

Current research on HIPP functions focuses primarily on model plants, with limited studies on maize. In this study, gene expression dynamics in shoots reveal that 83.33% (55) of *ZmHIPP* genes respond to Cd stress at 0 h, 12 h, and 72 h, including *ZmHIPP21*, *ZmHIPP09*, and *ZmHIPP02* (Figure 8). *ZmHIPP22* functions as a hub gene conferring Pb tolerance in maize seedlings [27]. Knockout of *ZmHIPP22* significantly inhibits the growth of maize seedlings under Pb stress and reduces Pb accumulation by blocking cellular uptake rather than facilitating cell wall sequestration. *ZmHIPP22* expression shows significant down-regulation (nearly 18-fold) at 12 h of excess Cd exposure (Figure 8), indicating that *ZmHIPP22* mitigates Cd toxicity by reducing its accumulation in shoots. A comparative transcriptomic analysis in roots of maize and rice subjected to Cd stress reveals rapid upregulation of *ZmHIPP21*, *ZmHIPP09*, and *ZmHIPP02*, as well as their rice counterparts. Notably, *ZmHIPP02* and its homolog *OsHIPP36* exhibit the most pronounced transcriptional activation. Furthermore, similar to its rice ortholog *OsHIPP42*, the common stress-responsive gene *ZmHIPP21* participates in various abiotic stresses (drought, salt, and low temperature) [16]. Collectively, these induced *ZmHIPP* genes, such as *ZmHIPP02*, *ZmHIPP21*, and *ZmHIPP22*, play important roles in coping with Cd stress and can be used as valuable genetic resources for breeding Cd-tolerant maize in the future.

Cd enters root cells through transporter proteins. After entering the cytosol, it is chelated into the vacuole through transporters and stored as a complex, which reduces Cd mobility and transfer from roots to leaves, resulting in significantly higher Cd accumulation in roots than in the aboveground parts (Figure 10). Cd is transported to grains via the xylem-to-phloem pathway, posing significant risks to crop production and food safety due to excessive Cd accumulation [30]. Root metal transporters are crucial components in defense against soil metal toxicity. Numerous transporter families facilitating Cd movement have been identified, including NRAMP [39,40], HMA [41], ZIP [42], and ABC [43]. HIPPs are plant-specific metal chaperones involved in plant development and abiotic stress responses, with particular significance in heavy metal transport [44]. Maize faces concerns regarding Cd contamination. Identification and functional screening of *ZmHIPP* genes will provide a foundation for targeted breeding of Cd-tolerant maize varieties and soil ecological remediation.

## 5. Conclusions

This study comprehensively characterized the *HIPP* family in maize and identified 66 *ZmHIPP* genes, which were divided into five groups and distributed across ten chromosomes. *ZmHIPP* genes are particularly conserved in evolution and closely related to orthologs in rice. Most *ZmHIPP* genes exhibit tissue-specific expression patterns during development, and 53 *ZmHIPP* genes were induced by excess Cd exposure. qRT-PCR analysis confirmed that six induced *ZmHIPPs* coordinated in response to Cd stress as they functioned in different sections and stages. The accumulation of Cd in roots was significantly greater than in shoots. Cd levels in roots increased until 120 h post-treatment, while the accumulation in shoots peaked at 72 h. This study includes a genome-wide assessment of the *HIPP* gene family in maize and offers critical insights for functional analyses of Cd stress in maize, suggesting a potential strategy for breeding Cd-tolerant maize and phytoremediation of Cd-contaminated soils.

## Data Availability

The data presented in this study are available in the Supplementary Materials.

## Supplementary Materials

The following supporting information can be downloaded at: https://www.mdpi.com/article/10.3390/genes16070770/s1, Table S1: Primers used in qRT-PCR; Table S2: The sequence information and physicochemical properties of ZmHIPPs.

## Abbreviations

The following abbreviations are used in this manuscript:

As	Arsenic
Cd	Cadmium
Cu	Copper
FPKM	Fragments Per Kilobase of transcript per Million mapped reads
GFF	General Feature Format
GTF	Gene Transfer Format
HIPP	Heavy metal-associated isoprenylated plant protein
HMA	Heavy metal-associated
HMM	Hidden Markov model
Pb	Lead
STRE	Stress response promoter element
